# Alexithymia and facial emotion recognition in patients with craniofacial pain and association of alexithymia with anxiety and depression: a systematic review with meta-analysis

**DOI:** 10.7717/peerj.12545

**Published:** 2021-11-29

**Authors:** Roy La Touche, Alberto García-Salgado, Ferran Cuenca-Martínez, Santiago Angulo-Díaz-Parreño, Alba Paris-Alemany, Luis Suso-Martí, Aida Herranz-Gómez

**Affiliations:** 1 Motion in Brains Research Group, Institute of Neuroscience and Sciences of the Movement (INCIMOV), Centro Superior de Estudios Universitarios La Salle, Universidad Autónoma de Madrid, Madrid, Spain; 2 Instituto de Dolor Craneofacial y Neuromusculoesquelético (INDCRAN), Madrid, Spain; 3 Departamento de Fisioterapia, Centro Superior de Estudios Universitarios La Salle, Universidad Autónoma de Madrid, Madrid, Spain; 4 Facultad de Medicina, Universidad CEU San Pablo, Madrid, Spain; 5 Departamento de Fisioterapia, Universidad CEU Cardenal Herrera, CEU Universities, Valencia, Spain

**Keywords:** Alexithymia, Craniofacial pain, Facial emotion recognition, Temporomandibular disorders, Depression, Anxiety

## Abstract

**Background:**

We aimed to determine the presence of alexithymia in patients with craniofacial pain (CFP) compared with asymptomatic individuals. Our secondary aims were to assess the relationship of alexithymia with anxiety and depression levels, as well as to assess the presence of facial emotion recognition deficit.

**Methods:**

Medline, Scielo and Google Scholar were searched, with the last search performed in 8 September 2021. Standardized mean differences (SMDs) and 95% CIs were calculated for relevant outcomes and were pooled in a meta-analysis using the random effects model. In addition, meta-analyses of correlations and a meta-regression of alexithymia with depression and anxiety were performed.

**Results:**

Regarding alexithymia, assessed through the Toronto Alexithymia Scale (TAS), the results showed significant differences, with higher values in patients compared with asymptomatic individuals, with a large clinical effect (SMD 0.46; 95% CI [0.22–0.71]; heterogeneity-Q 66.86; *p* < 0.001; inconsistency (I^2^) = 81%). We found statistically significant correlations with a small clinical effect of alexithymia with anxiety and depression. The meta-regression showed no significant association between the TAS and anxiety or depression. With respect to facial emotion recognition, the results showed statistically significant differences, with greater recognition difficulty in patients compared with asymptomatic individuals, with a large clinical effect (SMD −1.17; 95% CI [−2.01 to −0.33]; heterogeneity-Q 2.97; *p* = 0.080; I^2^ = 66%).

**Conclusions:**

Patients with CFP showed alexithymia with moderate evidence. There was also moderate evidence indicating that these patients had significant deficits in facial emotion recognition compared with asymptomatic individuals. Furthermore, alexithymia showed statistically significant correlations with anxiety and depression levels.

## Introduction

Craniofacial pain (CFP) is characterized by the presence of pain associated with disorders involving osteoarticular tissues of the cranio-orofacial region, including the teeth and their associated structures, and the temporomandibular joint and head region. Painful craniofacial conditions maintained over time represent a challenge in terms of clinical management (Chichorro, Porreca & Sessle, 2017).

Temporomandibular disorders (TMDs) and headaches are two of the most common CFP disorders. TMDs are characterized by pain that can be felt in the masticatory muscles, periauricular region, teeth and temporomandibular joint (Schiffman et al., 2014). TMDs are the primary cause of nonodontogenic orofacial pain, with considerable prevalence and chronicity rates, involving high levels of disability and socioeconomic costs (Conti et al., 2012). In addition, primary headaches are a global health concern, especially among women, with a prevalence of 30.8% for tension-type headaches and 18.1% for migraines, with a lower prevalence in men (Stovner et al., 2018). Primary headaches are the most common headaches, and are also frequently found in patients with TMD symptoms (Stovner et al., 2007; Glaros, Urban & Locke, 2007; Silveira et al., 2015).

Psychosocial factors also have an important association with the duration of symptoms and their perpetuation in cases of chronic pain, including patients with TMDs and headaches (Maísa Soares & Rizzatti-Barbosa, 2015). Individual differences in cognitive and emotional factors involved in the chronic pain experience could be related to the pain’s intensity and chronicity; thus, identifying biopsychosocial factors appears critical to improving the assessment and treatment of patients with TMDs and headaches (Gatchel et al., 2007).

One of the studied factors has been alexithymia, defined as the inability to label and describe emotions and a preference for externally oriented thinking (Sifneos, 1973). Patients with alexithymia have a cluster of cognitive and emotional traits, including difficulty identifying feelings, difficulty describing their feelings to others and a limited imaginative capacity (Taylor & Bagby, 2004). Alexithymia has been associated with poor recognition of facial emotions, which could involve a disruption of cortical motor processing (Gil et al., 2009).

Previous studies have shown a higher prevalence of clinical conditions as well as chronic pain in patients with alexithymia (Taylor, 2000; Fresán et al., 2021). A recent meta-analysis had found that alexithymia is shown to be elevated in people with chronic pain and is associated with pain intensity and physical function (Aaron et al., 2019). Although the mechanisms linking chronic pain with alexithymia remain uncertain, it appears that both physiological and behavioral mechanisms might be involved (Kano & Fukudo, 2013). The relationship between these aspects highlights the importance of understanding the role of alexithymia in patients with TMDs and headaches, which could improve the management of these patients from a biopsychosocial point of view.

CFP has a unique characteristic compared with other pain locations: the structures where the pain is felt is related to the function of those structures for emotional expression. It has been observed that patients with chronic CFP usually present with reduced facial movements, and they have difficulty recognizing their own emotions (Neal & Chartrand, 2011). A recent study has demonstrated how patients with migraine showed increased neural activation to fearful and happy faces, and linked the activation in the S1 and dorsal striatum to migraine frequency, concluding that sensitivity to emotional stimuli might play a role in triggering the headache (Szabó et al., 2019). We need to improve our understanding of the possible associations between alexithymia, emotion recognition and emotional expression in these patients.

Although the relationship between alexithymia and pain intensity is not yet clear, some studies have reported an association with depression levels (Di Tella & Castelli, 2016), finding higher levels of depression and pain intensity in patients with alexithymia compared with non-alexithymic patients with somatoform disorders (Probst et al., 2017). The presence of alexithymia has been found to correlate with high levels of anxiety and depression in patients with chronic migraine (Yalug et al., 2010). Also, alexithymia can act as a predictor variable for high levels of anxiety and depression in the general population (Honkalampi et al., 2000; Berardis et al., 2008).

Therefore, the main aim of the present systematic review and meta-analysis was to determine the presence of alexithymia in patients with CFP compared with asymptomatic individuals. The secondary aim was to assess whether there was a correlation of alexithymia with anxiety or depression, as well as to evaluate the presence of a deficit in facial emotion recognition in patients with craniofacial pain compared with asymptomatic individuals.

## Materials & methods

This systematic review and meta-analysis was performed in accordance with the Preferred Reporting Items for Systematic Reviews and Meta-analysis guidelines described by Moher et al. (2009). The protocol of this systematic review and meta-analysis was entered in an international register prior to starting the review (PROSPERO, CRD42020192261).

### Study selection

We included studies in the systematic review and meta-analysis if they met the following criteria: (1) an observational study with a cross-sectional, cohort or case-control design; (2) adult patients with CFP (headache or TMD) according to validated diagnostic criteria such as research diagnostic criteria for temporomandibular disorders or International Classification of Headache Disorders; (3) the degree of alexithymia and the facial discrimination of emotions was addressed and quantified; and (4) there was a control group for comparison.

### Search strategy

We conducted a search of observational and comparative studies that included cohort, case-control and cross-sectional studies using MEDLINE, Scielo and Google Scholar. The last search was performed on 8 September 2021.

The search strategy of each database can be found in Supplemental information. The search was conducted by two independent reviewers (RL and FCM) using the same methodology, and disagreements were resolved by consensus with a third reviewer (LSM). Reference lists from original studies were screened manually. Bibliographic references of identified publications and published bibliographic reviews were searched by hand for potentially relevant articles.

### Selection criteria and data extraction

Two independent reviewers (RL and FCM) performed the first phase: assessing the relevance of the studies. This first analysis was performed based on information from each study’s title, abstract and keywords. If the abstracts did not contain sufficient information, the full text was reviewed. During the second phase, we reviewed the full text and checked whether the studies met all of the inclusion criteria. A third reviewer (LSM) acted as mediator when there were differences between the 2 reviewers, with the 3 reviewers reaching a consensus (Furlan et al., 2009). The data described in the results were extracted by means of a structured protocol that ensured that the most relevant information from each study was obtained. To be able to perform the secondary analysis, data from the facial emotion recognition, anxiety and depression level variables were extracted from the studies that included them.

### Methodological quality assessment

We assessed the methodological quality of the selected cross-sectional studies, using a modified version of the Newcastle-Ottawa Quality Assessment Scale (NOS) adapted for cross-sectional studies (Deeks et al., 2003; Wells et al., 2009), which has moderate interrater reliability (*k* = 0.75, 95% CI [0.65–0.81]) (Zhang et al., 2021). The NOS scale ranges from 0 to 10 stars out of a total of seven questions within three sections: selection, comparability, and outcome. The tallied stars provide five categories of study quality: (1) poor, 0 to 3 stars; (2) fair, 4 to 5 stars; (3) good, 6 to 7 stars; (4) very good, 8 to 9 stars; and (5) excellent, 10 stars (Wells et al., 2009).

Two independent reviewers examined the quality of the selected studies using the same methods; disagreements between the reviewers were resolved by consensus, which included mediation by a third reviewer. The inter-rater reliability was determined using the Kappa coefficient: (1) κ > 0.7 indicated a high level of agreement between the assessors; (2) κ = 0.5–0.7 indicated a moderate level of agreement; and (3) κ < 0.5 indicated a low level of agreement (Cohen, 1960).

### Qualitative analysis

For the qualitative analysis of the selected cross-sectional studies, we employed an adaptation of the classification criteria provided by van Tulder et al. (2003) for randomized controlled trials. The results were categorized into 5 levels depending on the methodological quality: (1) strong evidence: consistent among multiple high-quality cross-sectional studies (at least 3); (2) moderate evidence: consistent findings from multiple good or very good-quality cross-sectional studies; (3) limited evidence: at least 2 fair quality cross-sectional studies; (4) conflicting evidence: inconsistent findings among multiple studies (cross-sectional studies); and (5) no evidence: no cross-sectional studies reported.

### Data synthesis and analysis

The statistical analysis was performed using meta-analyses with interactive explanations (MIX, version 1.7), with the data comparing patients with CFP and alexithymia with asymptomatic participants (Bax et al., 2006). We employed the same inclusion criteria for the systematic review and the meta-analysis, but added two criteria: (1) the results section contained detailed information on the comparative statistical data (mean, standard deviation and/or 95% confidence interval) of the Toronto Alexithymia Scale (TAS), either TAS-26 (Taylor et al., 1988) or TAS-20 (Bagby, Parker & Taylor, 1994); and (2) data for the analyzed variables were represented in at least two studies. Meta-analyses of correlations of alexithymia with anxiety or depression were also performed, including those studies that presented statistical data for alexithymia with anxiety or depression. We present the summary statistics in the form of forest plots (Lewis & Clarke, 2001), which consist of a weighted compilation of all standardized mean differences (SMDs) and corresponding 95% confidence intervals (CIs) reported by each study, and provide an indication of heterogeneity among the studies. The statistical significance of the pooled SMDs was examined using Hedges’ g, to account for possible overestimation of the true population effect size in small studies (Hedges, 1982). The magnitude of g was interpreted according to a 4-point scale: (1) <0.20, negligible effect; (2) 0.20‒0.49, small effect; (3) 0.50‒0.79, moderate effect; and (4) ≥0.80, large effect (Cohen, 1992). We estimated the degree of heterogeneity among the studies by employing Cochran’s Q statistic test (p <0.1 was considered significant) and the inconsistency index (I^2^) (Higgins et al., 2003): I^2^ > 25% is considered to represent low heterogeneity, I^2^ > 50% is considered medium, and I^2^ > 75% is considered to represent large heterogeneity (Huedo-Medina et al., 2006). The I^2^ index is complementary to the *Q* test, although it has a similar power problem as the *Q* test with a small number of studies (Huedo-Medina et al., 2006). Therefore, a study was considered heterogeneous when it fulfilled one or both of the following conditions: (1) the Q-test was significant (*p* < 0.1); and (2) the result of I^2^ was >75%. We performed a random-effects model in the meta-analysis of the heterogeneous studies to obtain a pooled estimate of effect (DerSimonian & Laird, 1986). To detect publication biases and to test the influence of each study, we performed a visual evaluation of the funnel plot and the exclusion sensitivity plot, searching for any asymmetry. We also employed Egger’s regression test to determine the presence of bias (Begg & Mazumdar, 1994; Sterne & Egger, 2001).

In addition, a meta-regression was performed using the OpenMEE program (Wallace et al., 2017) to assess whether Hedges’ g of alexithymia is linearly related to average normalized anxiety and depression. Previously, it was necessary to perform another meta-analysis including only the studies that assessed both the alexithymia variable and anxiety or depression.

## Results

**Figure 1:**
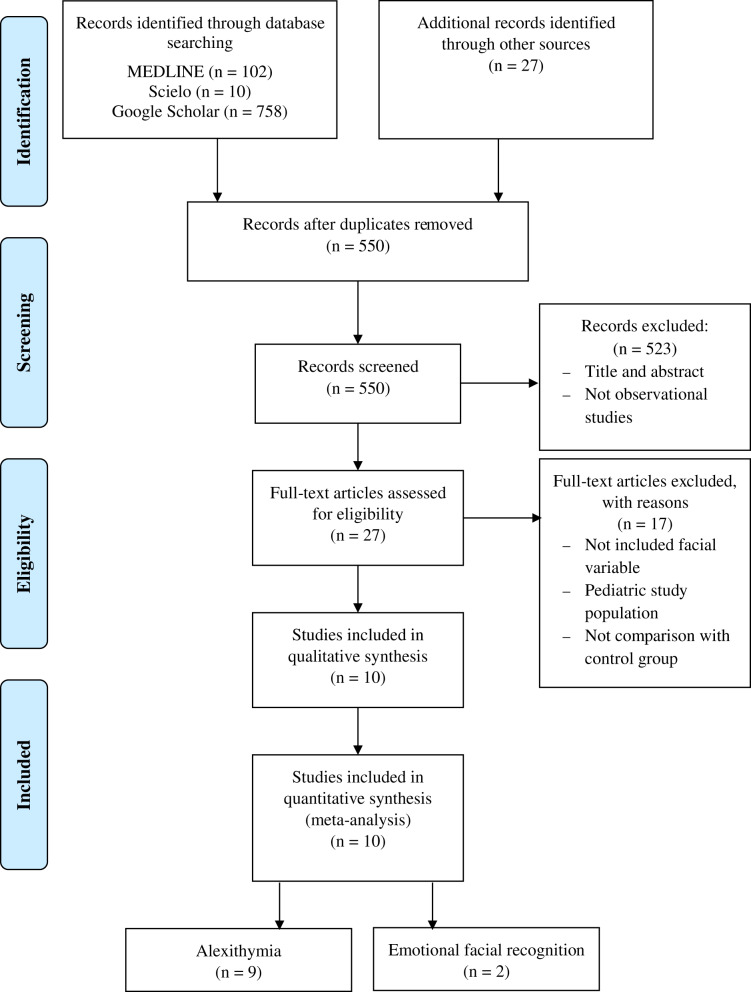
Flowchart diagram.

The study search strategy is presented in the form of a flow diagram (Fig. 1). A total of 10 cross-sectional studies met the inclusion criteria. Table 1 lists the epidemiological characteristics, the results and the conclusion of each article.

**Table 1:** Characteristics of included studies.

Article	Design	Sample characteristics of orofacial group	Sample characteristics of control group	Classification	Inclusion criteria	Measures	General conclusion
Glaros & Lumley (2005)	Cross-sectional	49 individuals with TMD	52 pain-free disk displacement controls or healthy individuals	–	Temporomandibular disorder pain, only people who receive the same diagnosis of both classifiers were included	TAS-20 total, TAS-DIF, TAS-DDF, TAS-EOT, SCL-90-R, pain in one week	Painful TMD had higher DIF, although this difference was fully explained by depressed mood. Global TAS-20, related to increased pain in the jaw
Galli et al. (2017)	Cross-sectional	Chronic migraine: 80 individuals (21 men and 59 women), age 44.65 (8.62) Episodic migraine without aura: 44 individuals (8 men and 36 women), age 42.18 (10.16)	67 healthy individuals (26 men and 41 women), age 41.21 (10.16)	ICHD-3 (beta-version)	Enrolled in Headache Science Center of the National Neurological Institute C. Mondino (Pavia, Italy) between May 2013 and June 2014	TAS-20 total, TAS-DIF, TAS-DDF, TAS-EOT	Patients with chronic migraine had higher scores on the TAS-DIF and TAS-total. Not significant differences between episodic and chronic migraine, even if the chronic migraine obtained higher scores in each factor TAS and total
Shim, Park & Park, 2018	Cross-sectional	Migraine: 91 individuals (16 men and 75 women), age 35.0 (1.3) Tensional headache: 32 individuals (8 men and 24 women), age 44.7 (14.7)	124 healthy individuals (25 men and 99 women), age 37.2 (12.4)	ICHD-2	Patients between 13 and 70 years old who visited a clinic of the Department of Neurology, with tension headache or migraine.	TAS-20 total, TAS-DIF, TAS-DDF, TAS-EOT, SCL-90-R, SF-8, HIT-6, VAS previous month and VAS actual.	Patients with tension headache had higher scores on TAS and TAS-DIF. The impact of the headache was correlated with the TAS.
Yücel et al. (2002)	Cross-sectional	105 individuals with tensional headache (23 men and 82 women), age 33 (10)	70 healthy individuals (16 men and 54 women), age 34 (9)	IHS diagnostic criteria	Patients evaluated with headache for the first time between 1998 and 2000 in a university pain clinic. Between 18 to 65 years, with at least primary education.	BDI, ATS, TAS-20.	The headache individuals had significantly higher scores on measures of depression, automatic thoughts and alexithymia and lower scores on assertiveness. Subjects with chronic tension-type headache had greater depression than those with episodic tension headaches.
Yalınay Dikmen et al. (2017)	Cross-sectional	145 individuals with migraine (34 men and 111 women), age 33.18 (8.6) Infrequent episodic migraine (*n* = 55) Frequent episodic migraine (*n* = 40) Chronic migraine (*n* = 50).	50 healthy individuals (16 men and 34 women), age 29.06 (7.6)	ICHD-3 (beta-version)	Patients enrolled in the Neurology Headache Maslak Hospital Clinic, Acıbadem University, between January and October 2016.	Duration and type of migraine, MIDAS, BDI, BAI, TAS-20 total, TAS-DIF, TAS-DDF, TAS-EOT	Anxiety was an important predictor of alexithymia, and it was related to depression. They found no difference between groups in TAS-Total.
Vieira et al. (2013)	Cross-sectional	40 women with migraine, age 43.58 (10.71).	33 women without migraine, age 42.15 (10.53)	ICHD-2	Women between 21 and 59 years old, registered in the Headache Unit of the Porto Alegre Clinic Hospital.	BDI, BAI, WHOQOL-BREF, VAS mean of the 3-last month.	The quality life of women with migraine was predicted by levels of depression and alexithymia factor (ability to express emotions and fantasies). Migraine group was composed of people with high anxiety, low quality life in the physical realm.
Muftuoglu et al. (2004)	Cross-sectional	50 individuals with migraine without aura (18 men and 32 women), age 32.1	50 healthy individuals (19 men and 31 women), age 29.8	IHS diagnostic criteria	At least primary education, over 15 years without psychiatric or chronic systemic diseases.	Frequency of 6-last months, BDI, STAI-I, STAI-II, TAS- 26.	Migraine were significantly more depressive, anxious, and more alexithymic than the control group, anxiety was significantly correlated with alexithymia in migraine.
von Piekartz et al. (2015)	Cross-sectional	19 individuals with chronic orofacial pain (4 men and 15 women), age 46.89 (13.33)	19 healthy individuals (4 men and 15 women), age 40.53 (11.31)	RDC-TMD	Between 18 to 65 years old, facial pain for the least 12 months.	Emotional facial recognition: precision and response time. BDI and pain intensity.	People with chronic facial pain made worse and take longer time to respond the recognition task.
Haas et al. (2013)	Cross-sectional	20 individuals with TMD (2 men and 18 women), age 40.6 (14.7)	20 healthy individuals (2 men and 18 women), age 41.2 (14.9)	RDC-TMD	Participants must be in pain at least 6 months classified as a temporomandibular disorder.	Precision facial emotion recognition, TAS-26, pain and HAMD.	Subjects with TMD had lower precision in the emotional recognition test with statistically significant differences, having higher alexithymia and depression scores.
Cerutti et al. (2016)	Cross-sectional	53 women with migraine, age 41.77 (3.92)	53 women without migraine, age 41.35 (5.31)	ICHD-3 (beta-version)	Patients with migraine diagnosis at least 6 months previously, without pharmacological prophylaxis	Headache Questionnaire (pain characteristics, frequency and period of headache, TAS-20, SCL-90-R	Alexithymia prevalence was more frequent in women with migraine compared to control group. In addition, women above the TAS-20 cut off presented higher mean scores on SCL-90-R, thus showing a relationship between migraine, alexithymia and psychopathological symptoms.

**Note:**

ATS: Automatic Thoughts Scale; BAI: Beck Anxiety Inventory; BDI: Beck Depression Inventory; HAMD: Hamilton Depression Rating Scale; HIT-6: Headache Impact Test-6; ICHD-3: International Classification of Headache Disorders, 3rd Edition; ICHD-2: International Classification of Headache Disorders, 2rd edition; IHS: International Headache Society; MIDAS: Migraine Disability Assessment Test; RDC-TMD: Research Diagnostic Criteria for Temporomandibular Disorder; SCL-90-R: Symptom Checklist-90-revised; SF-8: Short-Form Health survey; STAI: State-Trait Anxiety Inventory (STAI-I State Anxiety Inventory; STAI-II Trait Anxiety Inventory); TAS: Toronto Alexithymia Scale; TAS-20: 20-item Toronto Alexithymia Scale; TAS-26: 26-item Toronto Alexithymia Scale; TAS-DDF: Capacity to differentiate feelings and emotions; TAS-DIF: Capacity to describe feelings and emotions; TAS-EOT: Capacity to express outwardly oriented feelings; TMD: Temporomandibular Disorders; VAS: Visual Analogue Scale; WHOQOL-BREF: World Health Organization Quality of Life. Mean age data are presented as mean (standard deviation).

Ten articles had been included in five separate meta-analyses. The first meta-analysis included nine articles and assessed the degree of alexithymia in patients with CFP using the total TAS-20 (Glaros & Lumley, 2005; Cerutti et al., 2016; Galli et al., 2017; Shim, Park & Park, 2018; Yalınay Dikmen et al., 2020) and TAS-26 (Yücel et al., 2002; Muftuoglu et al., 2004; Vieira et al., 2013; Haas et al., 2013). The second, third and fourth meta-analyses included 6 articles each, and dealt with the TAS subscales (Glaros & Lumley, 2005; Vieira et al., 2013; Cerutti et al., 2016; Galli et al., 2017; Yalınay Dikmen et al., 2017; Shim, Park & Park, 2018). Finally, the fifth meta-analysis included 2 articles and evaluated facial emotion recognition (Haas et al., 2013; von Piekartz et al., 2015).

The meta-analysis of correlations between alexithymia and anxiety included 3 studies (Vieira et al., 2013; Yalınay Dikmen et al., 2017; Shim, Park & Park, 2018), while the correlation between alexithymia and depression used the same 3 studies, including an additional one (Glaros & Lumley, 2005).

The meta-regression analysis between the TAS and anxiety included 5 studies (Muftuoglu et al., 2004; Vieira et al., 2013; Cerutti et al., 2016; Yalınay Dikmen et al., 2017; Shim, Park & Park, 2018); the analysis between the TAS and depression included 3 additional studies (Yücel et al., 2002; Glaros & Lumley, 2005; Haas et al., 2013). The meta-regression between TAS-DIF and anxiety included 4 studies (Vieira et al., 2013; Cerutti et al., 2016; Yalınay Dikmen et al., 2017; Shim, Park & Park, 2018), and that between TAS-DIF and depression included one additional study (Glaros & Lumley, 2005).

### Characteristics of the study population

A total of 1,228 participants with CFP were included in this study, of which the experimental group had TMD pain in two studies (Glaros & Lumley, 2005; Haas et al., 2013), six studies reported migraine (Muftuoglu et al., 2004; Vieira et al., 2013; Cerutti et al., 2016; Galli et al., 2017; Yalınay Dikmen et al., 2017; Shim, Park & Park, 2018) and two reported tension-type headaches (Yücel et al., 2002; Shim, Park & Park, 2018). Within the studies on migraines, all subdivided the sample according to type (chronic-episodic) except four studies, which generalized the sample with the name migraine (Muftuoglu et al., 2004; Vieira et al., 2013; Cerutti et al., 2016; Shim, Park & Park, 2018).

Within the total sample, 856 participants were women, representing 100% of the sample in two studies (Vieira et al., 2013; Cerutti et al., 2016); in the rest, the sample varied between 82.4% and 62% (Yücel et al., 2002; Muftuoglu et al., 2004; Glaros & Lumley, 2005; Galli et al., 2017; Yalınay Dikmen et al., 2017; Shim, Park & Park, 2018), and in 1 study, the female sample was 90% (Haas et al., 2013). The average age of the sample was 38.71 years, and the average age ranged from 29.06 to 46.89 years.

### Results of the methodological quality

The agreement between the two evaluators according to the Kappa coefficient was high (κ = 0.72). The intervention of a third evaluator was necessary to achieve consensus on the quality of three studies.

With respect to the studies included, the average score was 7.40 ± 0.52. All studies showed very good methodological quality (7 or 8 stars) (Table 2).

**Table 2:** Analysis of the methodological quality of the studies included using the Newcastle-Ottawa Quality Assessment Scale (NOS).

Studies	Selection				Comparability	Outcome		Total
	Representativeness of the sample	Sample size	Non-respondents	Ascertainment of the exposure	Comparability	Assessment of the outcome	Statistical test	
Cerutti et al. (2016)			★	★★	★★	★	★	**7**
Galli et al. (2017)	★		★	★★	★★	★	★	**8**
Glaros & Lumley (2005)	★		★	★★	★	★	★	**7**
Haas et al. (2013)			★	★★	★★	★	★	**7**
Muftuoglu et al. (2004)			★	★★	★★	★	★	**7**
Shim, Park & Park, 2018			★	★★	★★	★	★	**7**
Vieira et al. (2013)	★		★	★★	★★	★	★	**8**
von Piekartz et al. (2015)			★	★★	★★	★	★	**7**
Yalınay Dikmen et al. (2017)	★		★	★★	★★	★	★	**8**
Yücel et al. (2002)	★		★	★★	★★	★	★	**8**

**Note:**

★ = Present; no star = not present.

### Evidence of alexithymia in craniofacial pain

Nine studies analyzed the variable of alexithymia (Yücel et al., 2002; Muftuoglu et al., 2004; Glaros & Lumley, 2005; Vieira et al., 2013; Haas et al., 2013; Cerutti et al., 2016; Galli et al., 2017; Yalınay Dikmen et al., 2017; Shim, Park & Park, 2018) in patients with CFP (migraine, headache and temporomandibular disorder) *vs* healthy individuals using the TAS self-registration tool. In all the studies, significant differences between the symptomatic groups and the control group were reported, declaring higher scores in the total TAS.

Two studies assessed patients with tension headache, the study by Yücel et al. (2002) observing that there were no significant differences between headache groups (episodic and chronic). Another study had found that the TAS scores were higher for the tension headache groups than for the migraine group, which also had higher pain intensity scores, correlating the impact of the headache with the total TAS (Shim, Park & Park, 2018). In 5 studies, the comparison group were patients with migraine (Muftuoglu et al., 2004; Vieira et al., 2013; Galli et al., 2017; Yalınay Dikmen et al., 2017; Shim, Park & Park, 2018), and the only study whose results differed from the rest was that by Yalınay Dikmen et al. (2017), who reported no significant differences between the 2 groups, even with the control group having higher mean scores than the sum of the total migraine group scores. For the migraine studies that assessed anxiety, this variable correlated with high levels of alexithymia, whereas depression was not correlated with this as a predictor variable (Muftuoglu et al., 2004; Vieira et al., 2013; Yalınay Dikmen et al., 2017; Shim, Park & Park, 2018); however, when added to the previous variables it did predict the individuals’ quality of life (Vieira et al., 2013). Cerutti et al. (2016) found significant differences between women with and without alexithymia for both depression and anxiety.

Finally, only two studies assessing individuals with TMDs had concluded that high TAS values were significant compared with the healthy group, directly correlating higher scores with greater pain intensity and greater depression levels (Glaros & Lumley, 2005). Haas et al. compared the TAS with facial emotion recognition in images, obtaining an inverse relationship between higher TAS levels with lower emotional recognition capacity (Haas et al., 2013).

There are three factors that subdivide the TAS tool (TAS-20 and TAS-26): the capacity to differentiate feelings and emotions (DDF), the capacity to describe feelings and emotions (DIF) and the capacity to express outwardly oriented feelings (EOT). It is important to note that the DDF provides much more information compared with the other factors, given that higher scores and statistically significant differences have been observed in relation to healthy individuals (Glaros & Lumley, 2005; Cerutti et al., 2016; Galli et al., 2017; Shim, Park & Park, 2018), this factor correlating only with pain severity. However, when controlling for the depression variable, the DDF changes to EOT (Glaros & Lumley, 2005). On the other hand, among symptomatic groups, individuals with tension headache obtain higher DIF and EOT scores, as shown in one study (Shim, Park & Park, 2018), just as there are significant differences in the DIF among migraine groups, with the less frequent temporal attack groups being those who report higher scores (Yalınay Dikmen et al., 2017). Finally, the study by Vieira et al. reveals a correlation with the total TAS as a predictive factor for quality of life. This study also includes EOT as a predictor for individuals with migraine and highlights the fact that for healthy groups, it is the DIF in the correlation that predicts the individuals’ quality of life (Vieira et al., 2013).

### Quantitative analysis

#### TAS

**Figure 2:**
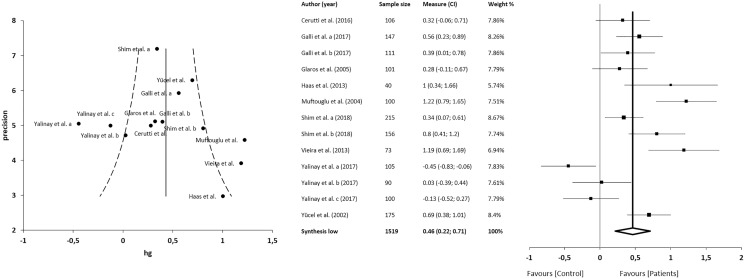
Synthesis forest and funnel plot of the TAS variable. The forest plot summarises the results of included studies (sample size, standardised mean differences (SMDs) and weight). The small boxes with the squares represent the point estimate of the effect size and sample size. The lines on either side of the box represent a 95% confidence interval (CI). Funnel plot aims to assess the existence of publication bias.

The meta-analysis showed statistically significant differences, with higher values in patients compared with asymptomatic individuals, with a small clinical effect in 9 studies (*n* = 1519; SMD 0.46; 95% CI [0.22–0.71]; heterogeneity *Q* value 66.86; *p* < 0.001; inconsistency (I^2^) = 81%, 95% CI [69–89]). Egger’s test results suggested no significant evidence of publication bias for the TAS analysis (intercept = 3.28; *t* = 0.66; *p* = 0.53). The shape of the funnel plot appeared to be symmetrical in the dominant model (Fig. 2). The sensitivity exclusion analysis suggested that no study significantly affected the pooled SMD (Fig. S1).

#### TAS-DDF

**Figure 3:**
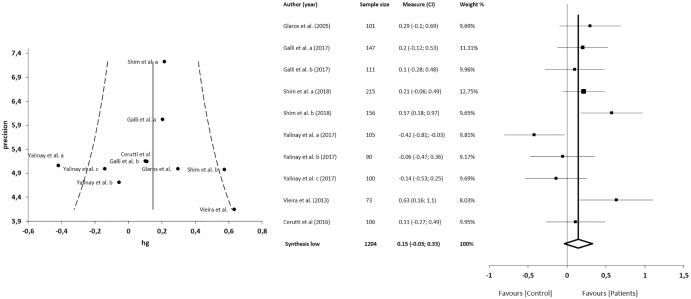
Synthesis forest and funnel plot of the TAS-DFF subscale (capacity to differentiate feelings and emotions). The forest plot summarises the results of included studies (sample size, standardised mean differences (SMDs) and weight). The small boxes with the squares represent the point estimate of the effect size and sample size. The lines on either side of the box represent a 95% confidence interval (CI). The funnel plot aims to assess the existence of publication bias.

The meta-analysis showed no statistically significant differences in 6 studies (*n* = 1,204; SMD 0.15; 95% CI [−0.03 to 0.33]; heterogeneity *Q* value 20.76; *p* = 0.01; I^2^ = 57%, 95% CI [12–79]). Egger’s test results suggested no significant evidence of publication bias for the analysis of TAS-DDF (intercept = 3.19; *t* = 0.00; *p* = 1). The shape of the funnel plot appeared to be symmetrical in the dominant model (Fig. 3). The sensitivity exclusion analysis suggested that no study significantly affected pooled SMD (Fig. S1).

#### TAS-DIF

**Figure 4:**
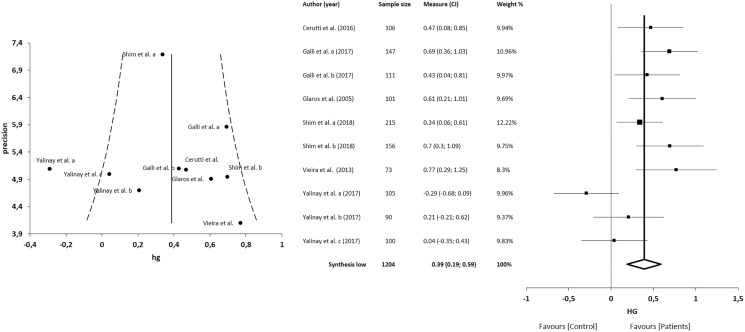
Synthesis forest and funnel plot of the TAS-DIF subscale (capacity to describe feelings and emotions). The forest plot summarises the results of included studies (sample size, standardised mean differences (SMDs) and weight). The small boxes with the squares represent the point estimate of the effect size and sample size. The lines on either side of the box represent a 95% confidence interval (CI). The funnel plot aims to assess the existence of publication bias.

The meta-analysis showed statistically significant differences, with higher values in patients compared with asymptomatic individuals, with a large clinical effect in 6 studies (*n* = 1204; SMD 0.39; 95% CI [0.19–0.59]; heterogeneity *Q* value 25.17; *p* < 0.001; I^2^ = 64%, 95% CI [30–82]). Egger’s test results suggested no significant evidence of publication bias for the analysis of the TAS-DIF (intercept = 3.80; *t* = 0.23; *p* = 0.82). The shape of the funnel plot appeared to be symmetrical in the dominant model (Fig. 4). The sensitivity exclusion analysis suggested that no study significantly affected the pooled SMD (Fig. S2).

#### TAS-EOT

**Figure 5:**
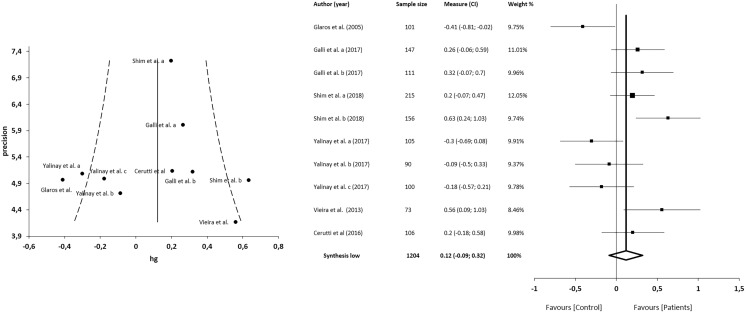
Synthesis forest and funnel plot of the TAS-EOT subscale (capacity to express outwardly oriented feelings). The forest plot summarises the results of included studies (sample size, standardised mean differences (SMDs) and weight). The small boxes with the squares represent the point estimate of the effect size and sample size. The lines on either side of the box represent a 95% confidence interval (CI). The funnel plot aims to assess the existence of publication bias.

The meta-analysis showed no statistically significant differences in 5 studies (*n* = 1204; SMD = 0.12; 95% CI [−0.09 to 0.32]; heterogeneity *Q* value = 26.81; *p* < 0.001; I^2^ = 66%, 95% CI [34–83]). Egger’s test results suggested no significant evidence of publication bias for the analysis of TAS-EOT (intercept = 3.92; *t* = −0.22; *p* = 0.83). The shape of the funnel plot appeared to be symmetrical in the dominant model (Fig. 5). The sensitivity exclusion analysis suggested that no study significantly affected the pooled SMD (Fig. S2).

#### Correlations between alexithymia and anxiety

**Figure 6:**
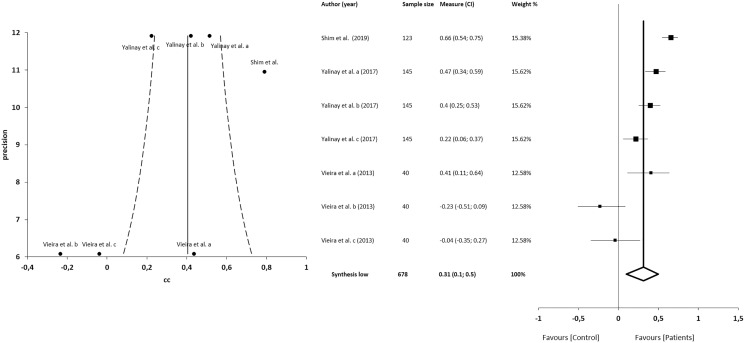
Synthesis forest and funnel plot of the correlation between alexithymia and anxiety. The forest plot summarises the results of included studies (sample size, standardised mean differences (SMDs) and weight). The small boxes with the squares represent the point estimate of the effect size and sample size. The lines on either side of the box represent a 95% confidence interval (CI). Funnel plot aims to assess the existence of publication bias.

The meta-analysis for the association between alexithymia and anxiety in patients with CFP showed statistically significant correlations with a small clinical effect in 3 studies (*n* = 678; SMD 0.31; 95% CI [0.10–0.50]; heterogeneity *Q* value 46.59; *p* < 0.001; I^2^ = 87%, 95% CI [76–93]). Egger’s test results suggested no significant evidence of publication bias for the analysis of alexithymia and anxiety (intercept = 2.52; *t* = −2.06; *p* = 0.09). The shape of the funnel plot appeared to be asymmetrical in the dominant model (Fig. 6). The sensitivity exclusion analysis suggested that no study significantly affected the pooled SMD (Fig. S3).

#### Correlations between alexithymia and depression

**Figure 7:**
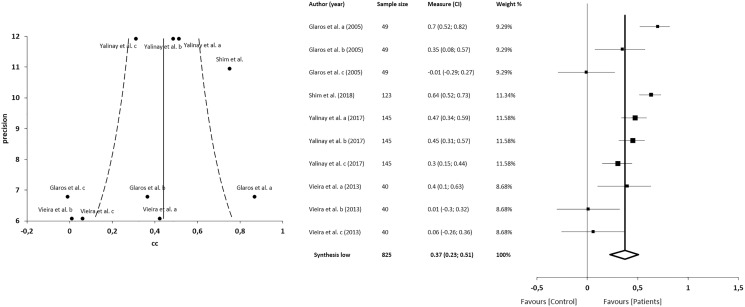
Synthesis forest and funnel plot of the correlation between alexithymia and depression. The forest plot summarises the results of included studies (sample size, standardised mean differences (SMDs) and weight). The small boxes with the squares represent the point estimate of the effect size and sample size. The lines on either side of the box represent a 95% confidence interval (CI). Funnel plot aims to assess the existence of publication bias.

The meta-analysis for the association between alexithymia and depression in patients with CFP showed statistically significant correlations with a small clinical effect in 4 studies (*n* = 825; SMD 0.37; 95% CI [0.23–0.51]; heterogeneity *Q* value 45.21; *p* < 0.001; I^2^ = 80%, 95% CI [64–89]), Egger’s test results suggested no significant evidence of publication bias for the analysis of alexithymia and depression (intercept = 1.82; *t* = −1.94; *p* = 0.09). The shape of the funnel plot appeared to be symmetrical in the dominant model (Fig. 7). The sensitivity exclusion analysis suggested that no study significantly affected the pooled SMD (Fig. S3).

#### Meta-regression between alexithymia and anxiety or depression

**Figure 8:**
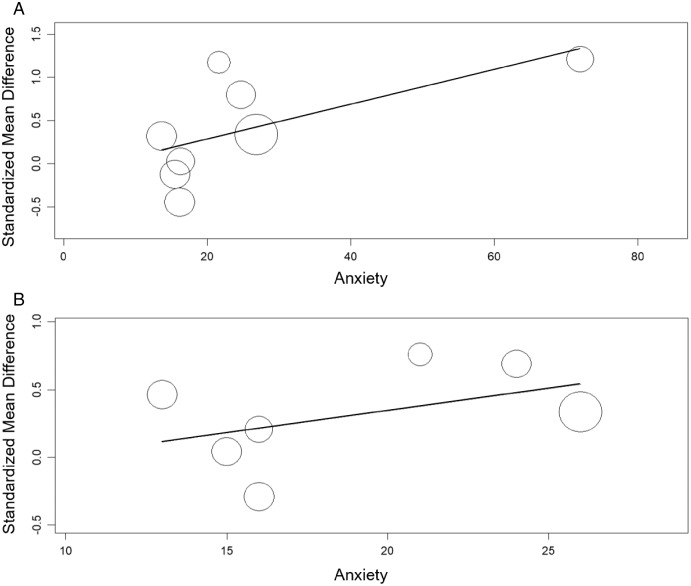
Meta-regression analysis for the effect of anxiety on the alexithymia: TAS variable and anxiety (A) and, TAS-DIF subscale (capacity to describe feelings and emotions) and anxiety (B).

**Figure 9:**
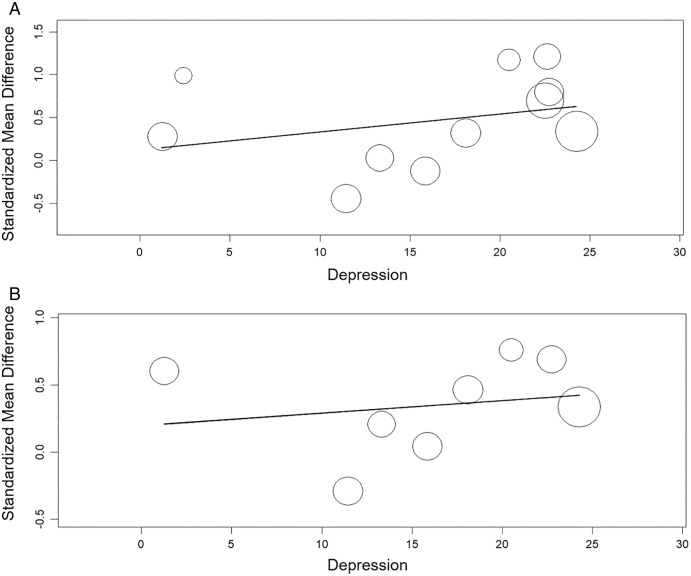
Meta-regression analysis for the effect of depression on the alexithymia: TAS variable and depression (A) and, TAS-DIF subscale (capacity to describe feelings and emotions) and depression (B).

The meta-regression showed significant relation between the TAS and anxiety (β = 0.020, 95% CI [0.003 to 0.04], *p*< 0.05) nut not between the TAS and depression (β = 0.021, 95% CI [−0.02 to 0.06], *p* > 0.05). The meta-regression showed no significant relation between the TAS-DIF and anxiety or depression (β = 0.033, 95% CI [−0.02 to 0.09], *p* > 0.05 and β = 0.009, 95% CI [−0.03 to 0.05], *p* > 0.05, respectively) (Figs. 8 and 9).

### Facial emotion recognition

The facial emotion recognition test consists of presenting a series of images to the participants that show a face expressing the 6 basic emotions (happiness, sadness, anger, fear, disgust and surprise), as well as a neutral addition that they should identify as they see fit. Only 2 studies (Haas et al., 2013; von Piekartz et al., 2015) compared CFP (in this case TMDs) with asymptomatic individuals and with a transversal character. In both, individuals with temporomandibular pain were less accurate in identifying the presentation of images. In the Haas et al. study, participants identified significantly fewer emotions of sadness and fear, and the emotion of disgust was incorrectly identified significantly more often. For anger, happiness, disgust and surprise they found no statistically significant differences between groups (Haas et al., 2013). von Piekartz et al. (2015) had also documented the response time of the participants, finding that individuals in pain took longer to respond. Happiness, disgust and anger were more accurately identified than surprise, and disgust more accurately than sadness.

#### Quantitative analysis

**Figure 10:**
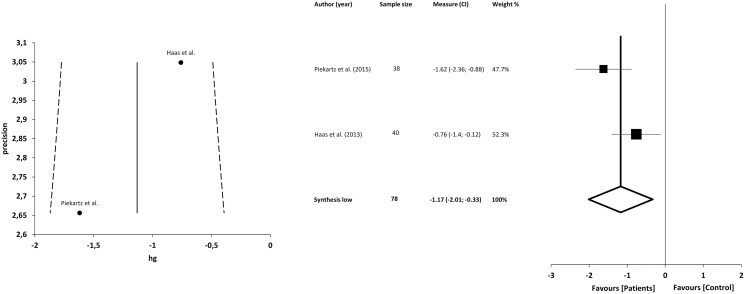
Synthesis forest and funnel plot of emotion facial recognition. The forest plot summarises the results of included studies (sample size, standardised mean differences (SMDs) and weight). The small boxes with the squares represent the point estimate of the effect size and sample size. The lines on either side of the box represent a 95% confidence interval (CI). Funnel plot aims to assess the existence of publication bias.

The meta-analysis showed statistically significant differences, with greater difficulty of recognition in patients with CFP compared with asymptomatic individuals, with a large clinical effect in 2 studies (*n* = 78; SMD −1.17; 95% CI [−2.01 to −0.33]; heterogeneity *Q* value 2.97; *p* = 0.08; I^2^ = 66%). The shape of the funnel plot appeared to be symmetrical in the dominant model (Fig. 10).

## Discussion

The main aim of the present systematic review and meta-analysis was to determine the presence of alexithymia in patients with CFP compared with asymptomatic individuals. The secondary aim was to assess whether there was a correlation between alexithymia and anxiety or depression levels, as well as to evaluate whether patients with CFP present a deficit in facial emotion recognition, also compared with asymptomatic individuals.

The results show, first, that patients with CFP show alexithymia in the TAS and TAS-DIF dimension compared with asymptomatic individuals, but not in the case of the DDF and EOT subscales. In addition, statistically significant correlations between alexithymia and anxiety and depression were found. A linear dependence was found only between the TAS and anxiety, but not in the case of depression. Furthermore, the results also showed significant deficits in facial emotion recognition in patients with CFP compared with asymptomatic individuals.

In the present review, the results of both TAS versions (20-items and 26-items) were used, given that both inventories significantly correlate with each other (Wise, Simpson & Sheridan, 2000). For the meta-analysis, however, only the common specific subscales of each version were regarded for the statics.

The results of the present study highlight the close relationship between CFP and the presence of alexithymia. In this respect, it appears that alexithymia could be associated with the biological dimension of chronic pain, given that some previous laboratory research has shown alterations in the processing of pain, both in asymptomatic individuals and in patients with fibromyalgia, suggesting a process of hypersensitivity to the sensory component of pain in these patients (Nyklíček & Vingerhoets, 2000; Huber et al., 2009).

In addition, alexithymia has been found to be associated with dysfunctions in the autonomic nervous system and the neuroendocrine system, which could overlap with the biological alterations of patients with chronic pain (Lumley, Neely & Burger, 2007). Research has shown that alexithymia is correlated with physical symptom overreporting, and its degree could be associated with aberrant pain intensity and sensitivity perception (Nakao et al., 2002).

However, it is not possible to explain the relationship between the presence of CFP and alexithymia only from a biological perspective. The experience of pain often involves the presence of cognitive-emotional aspects, such as anxiety or pain catastrophism (Giannakopoulos et al., 2010; Turk et al., 2016). In this regard, the amygdala has a fundamental role in emotional regulation, and people with alexithymia exhibit alterations in the activity of this structure in the processing of emotions (LeDoux, 2003; Reker et al., 2010).

As in the present study, (Aaron et al., 2019) also found no significant differences for the EOT subscale, which could be due to the fact that chronic pain is more related to the difficulty in identifying and describing feelings and emotions or in the cognitive component; they did find differences in the DDF subscale.

In addition, when correlating with pain intensity, alexithymia showed associations with depressive and anxiogenic symptoms, which could be due to the reduced ability to regulate negative emotions that it implies (Leweke et al., 2011; Aaron et al., 2019).

Specifically, it appears that the DIF subscale of the TAS was associated with and acted as a predictor of psychopathological factors, such as depression, whereas the DDF and EOT scales have a lesser influence (Conrad et al., 2009). In fact, an improvement in depressive symptoms in patients with depression was reflected in improved scores on the DIF and DDF scales, but not on the EOT subscale (Saarijärvi, Salminen & Toikka, 2006).

People with alexithymia could have poorer emotional regulation; thus, the presence of these added stressors could contribute to increased pain experiences and poorer health outcomes (Lumley, Beyer & Radcliffe, 2008). The relationships between the presence of CFP and alexithymia are based on socio-affective and neurobiological alterations, indicating that the lesser emotional processing and regulation, as well as the greater degree of activation or interoceptive sensitivity, are related to pain in these patients (Kano & Fukudo, 2013).

Another major finding of the present study is the lack of facial emotion recognition in patients with CFP. The recognition of facial emotions is strongly associated with alterations in motor processing, an aspect that has been reported in the literature in recent years, and also appears to be linked to the presence of chronic pain (Bray & Moseley, 2011). However, it is possible that the deficit of facial emotion recognition is not directly related to the presence of pain, but to the presence of alexithymia, which could be a better predictor of emotional recognition disruption (Haas et al., 2013).

The dorsal anterior cingulate cortex and bilateral insula were less activated in patients with alexithymia in response to angry faces compared with their responses to neutral faces, and the dysfunction in this region has been proposed to be one of the main mechanisms of alexithymia (Lane et al., 1997; Adolphs, 2002). These findings are in line with the results obtained in this area, but further research is needed into the alterations in motor and emotional processing in patients with CFP.

Alexithymia could be an example of inflexible emotional regulation, with dysfunctions both psychologically and physiologically. These maladaptive processes and their consequence on allostatic load potentially explain the association between alexithymia and physical and mental illness. It is suggested that a failure in anticipation using prior learning could be part of the alexithymia, and also early perceptual difficulties based on a possible poor early encoding, which could interfere in the processing and awareness of emotional stimuli. This hypo-reactivity has been shown in alexithymia through reduced physiological reactivity, as well as in depression and generalized anxiety (Panayiotou, Panteli & Vlemincx, 2021).

### Limitations

This systematic review with meta-analysis presents a number of limitations. First, the design of the studies prevents a cause-effect relationship from being established. Future studies using prospective longitudinal designs are needed to better understand how alexithymia influences CFP. Second, the quantitative results of the meta-analysis showed the presence of high heterogeneity, which should be taken into consideration as an important limitation. Third, the outcome measures are all based on self-reported registers. Fourth, it could be possible that the databases used for the research could generate a limitation, given that they are not the common ones; however, the authors wanted to guarantee that the research could be freely replicable, thus also included the Portuguese and Spanish language databases. Finally, one meta-analysis consisted of only 2 studies; thus, the findings should be interpreted with caution.

### Clinical implications and future research

The presence of alexithymia is related to biological dysfunctions and also to alterations in the psychosocial sphere. It is therefore that in the management of patients with CFP, the assessment of alexithymia should be considered clinically. Clinically, the deficit of facial emotion recognition might not be directly related to the presence of pain, but to the presence of alexithymia, which could be a better predictor of emotional recognition disruption. Future studies should aim to identify the associations between alexithymia with other variables and the timeline as well as calculating the odds ratio.

## Conclusions

The results of this systematic review and meta-analysis show, with moderate evidence, that patients with CFP show higher levels of alexithymia compared with participants without CFP. There was also moderate evidence indicating that these patients have significant deficits in facial emotion recognition compared with asymptomatic individuals. Finally, an association was shown between alexithymia and depression and anxiety levels.

## Supplemental Information

10.7717/peerj.12545/supp-1 Supplemental Information 1 Exclusion sensitivity plot for total TAS (A) score and TAS-DDF (B) subscale. A random-effects model was used.

10.7717/peerj.12545/supp-2 Supplemental Information 2 Exclusion sensitivity plot for total TAS-DIF (A) and TAS-EOT (B) subscales. A random-effects model was used.

10.7717/peerj.12545/supp-3 Supplemental Information 3 Exclusion sensitivity plot for the correlation between TAS variable and anxiety (A) and TAS variable and depression (B). A random-effects model was used.

10.7717/peerj.12545/supp-4 Supplemental Information 4 PRISMA checklist.

10.7717/peerj.12545/supp-5 Supplemental Information 5 Raw data.

10.7717/peerj.12545/supp-6 Supplemental Information 6 Search strategy.

## Additional Information and Declarations

### Data Availability

The following information was supplied regarding data availability: The raw data used for statistical analysis are available in the Supplemental File.
