# Supplementary figures and images for: Alexithymia and facial emotion recognition in patients with craniofacial pain and association of alexithymia with anxiety and depression: a systematic review with meta-analysis
Source: PeerJ. 2021 Nov 29;9:e12545. doi: 10.7717/peerj.12545 (PMC8638568; doi:10.7717/peerj.12545)

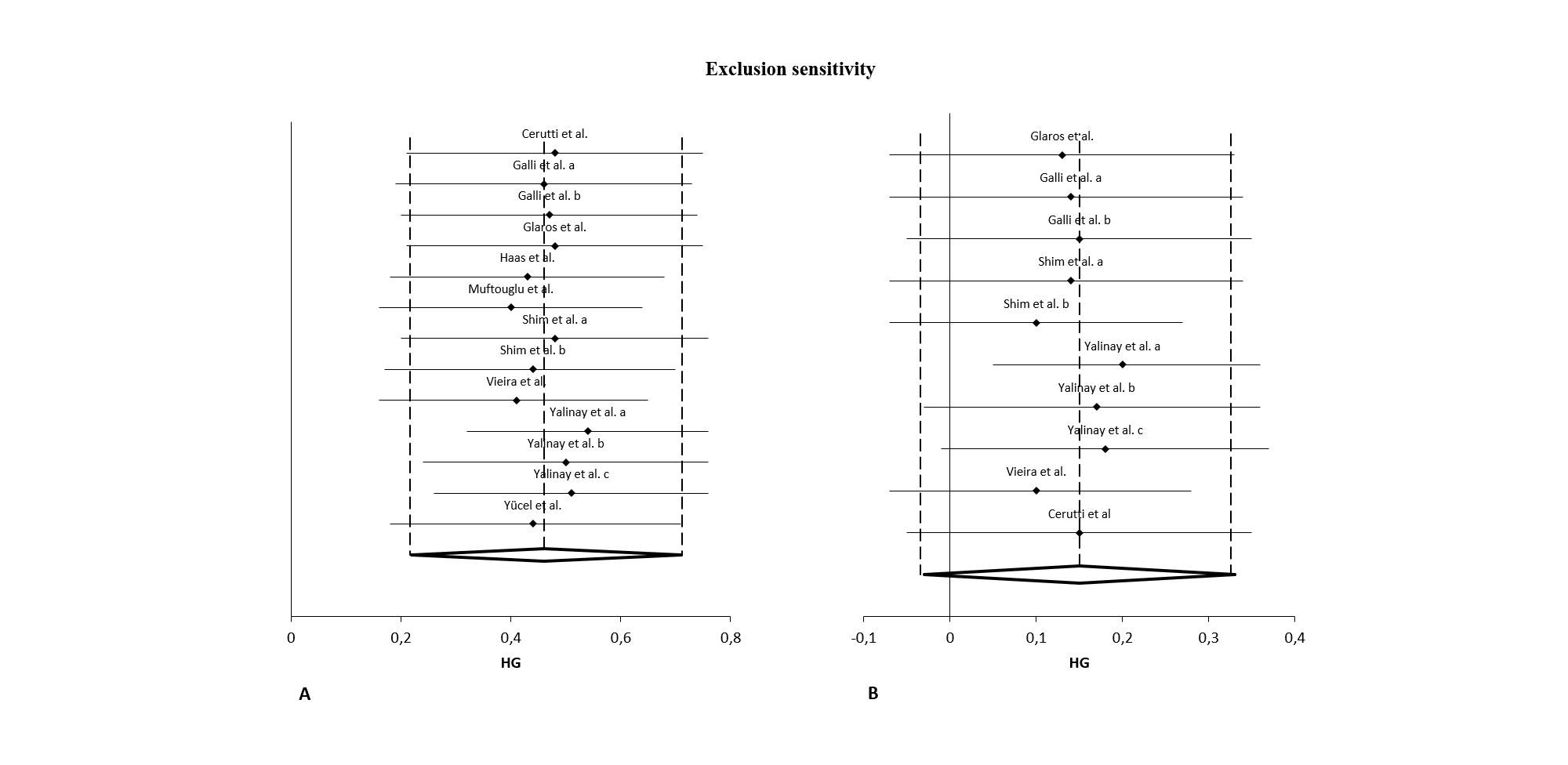

Supplement: Supplemental Information 1 [file peerj-09-12545-s001.png]

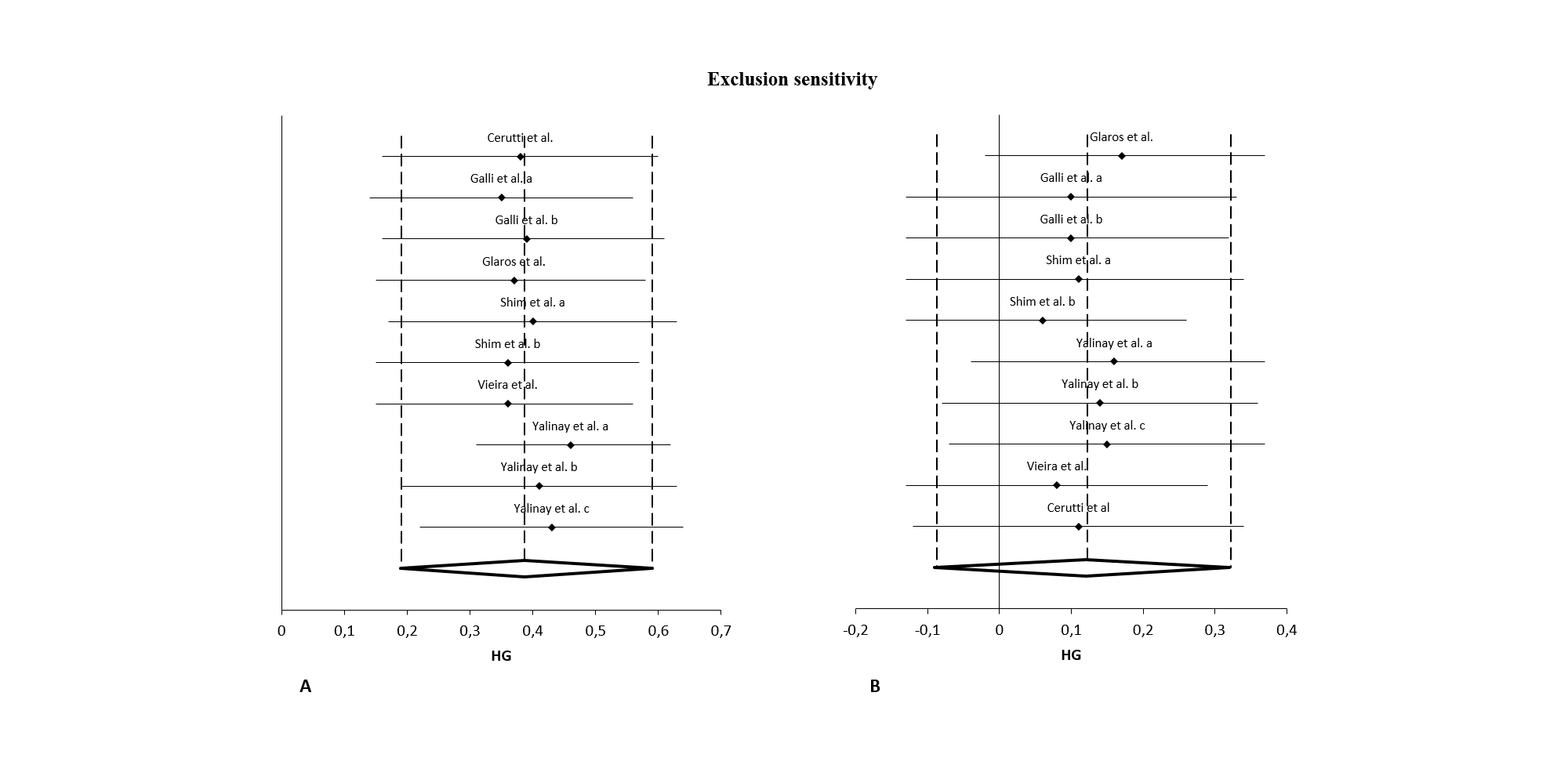

Supplement: Supplemental Information 2 [file peerj-09-12545-s002.png]

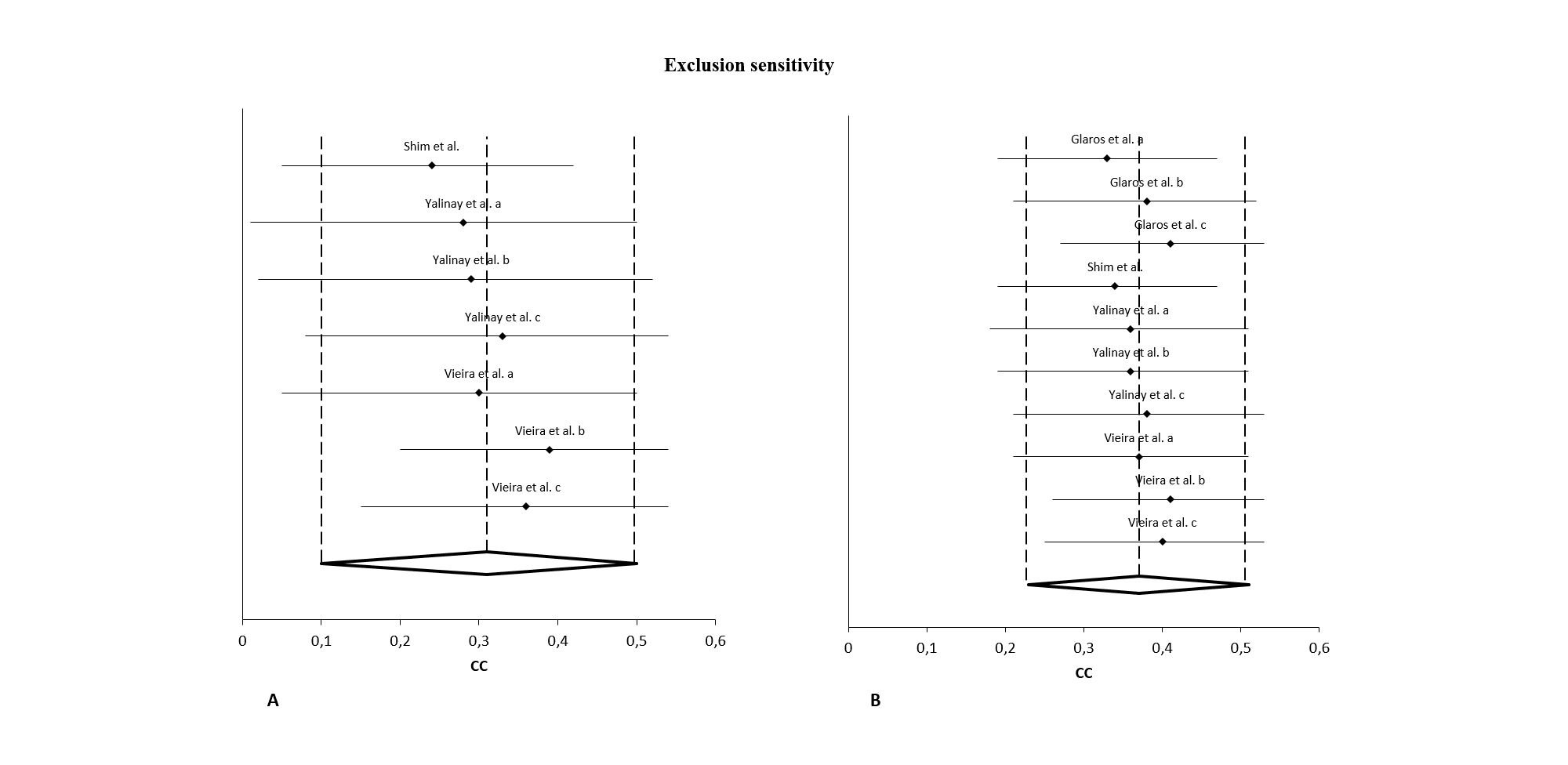

Supplement: Supplemental Information 3 [file peerj-09-12545-s003.png]
