# Supplementary material for: Alexithymia and facial emotion recognition in patients with craniofacial pain and association of alexithymia with anxiety and depression: a systematic review with meta-analysis
Source: PeerJ. 2021 Nov 29;9:e12545. doi: 10.7717/peerj.12545 (PMC8638568; doi:10.7717/peerj.12545)
Supplement: Supplemental Information 6 [file peerj-09-12545-s006.docx]

**Suplemmental information.** Search strategies.

***MEDLINE:***

((((((orofacial pain[MeSH Terms]) OR (craniofacial pain[MeSH Terms])) OR (headache)) OR (temporomandibular disorder)) AND ((facial emotion recognition) OR (alexithymia)))) AND (((observational) OR (cross-sectional)) OR (cohort))

***Scielo:***

((craniofacial pain) OR (orofacial pain) OR (headache) AND (temporomandibular disorder)) AND (alexithymia) OR (facial emotion recognition)

***Google Scholar:***

Filters: “With all the words”, “Throughout the article”

("craniofacial pain" OR "orofacial pain" OR "migraine" OR "temporomandibular disorder") AND ("facial emotion recognition" OR "alexithymia") AND ("observational" OR "cross-sectional" OR "cohort)
